# Performance of Daily Living Activities of Autistic Children With Sensory Processing Difficulties: Caregivers′ Perceptions

**DOI:** 10.1155/oti/5556699

**Published:** 2026-03-25

**Authors:** Luiza Salomão Tazinaffo, Bianca Brunelli Wolf, Amanda Mota Pacciulio Sposito

**Affiliations:** ^1^ Department of Health Sciences, Ribeirão Preto Medical School, University of São Paulo, Ribeirão Preto, São Paulo, Brazil, usp.br; ^2^ Municipal Health Department, Orlândia, São Paulo, Brazil

**Keywords:** activities of daily living, autism spectrum disorder, occupational therapy, sensory integration dysfunction, sensory processing

## Abstract

**Background:**

Sensory integration difficulties are common among autistic children and can significantly affect their occupational performance, particularly in activities of daily living (ADLs). Despite the increasing recognition of these challenges, few studies have specifically examined how sensory and praxis difficulties interfere with functional autonomy in daily routines.

**Objective:**

This study is aimed at analyzing caregivers′ perceptions of sensory processing difficulties and their impact on the performance of autistic children in basic ADLs.

**Methods:**

A qualitative, descriptive study was conducted with caregivers of children aged 3–8 years diagnosed with autism spectrum disorder (ASD), recruited from a private pediatric rehabilitation clinic in Brazil. Data were collected through semistructured interviews focusing on children′s engagement in and performance of ADLs. Interviews were audio‐recorded, transcribed verbatim, and analyzed using thematic content analysis with the support of MAXQDA software to systematically identify and categorize recurring sensory and motor‐related difficulties reported by caregivers.

**Results:**

Caregivers consistently reported that sensory processing difficulties had a significant negative impact on multiple domains of daily living activities, notably feeding, personal hygiene, dressing, bathing, and toileting, resulting in a restricted active and independent participation. Sensory modulation difficulties, including patterns of hyperresponsiveness and hyporesponsiveness, affected performance across all assessed activities. Sensory discrimination difficulties were particularly evident in dressing and toileting tasks. Praxis‐related difficulties were identified in all children and manifested as challenges in ideation, motor planning, and motor execution, which collectively compromised functional performance across daily activities and increased dependence on caregivers during daily routines.

**Conclusions:**

Sensory processing difficulties involving modulation, discrimination, and praxis substantially limit functional independence and occupational engagement in children with ASD. These findings highlight the importance of individualized occupational therapy interventions tailored to each child′s sensory and motor profile, with the aim of supporting participation, autonomy, and engagement in everyday activities.

## 1. Introduction

Autism spectrum disorder (ASD) is a neurodevelopmental condition characterized by deficits in social communication and restricted and repetitive patterns of behavior. The diagnosis is clinical and defined according to levels of required support [1]. According to the Centers for Disease Control and Prevention, the prevalence of ASD has increased significantly in recent years and is currently estimated at 1‐in‐31 8‐year‐old children in the United States [2].

Sensory processing abnormalities are highly prevalent in this population [2, 3], with a reported incidence as high as 96% [4], and are recognized as an important diagnostic criterion [5, 6]. Sensory systems can be classified into tactile, proprioceptive, vestibular, gustatory, auditory, olfactory, visual, and interoceptive domains. The integration of these systems is essential for daily functioning, as sensory stimuli are constantly present and simultaneously processed [7].

Given this complexity, Jean Ayres proposed, in 1970, the sensory integration theory, defining it as a neurological process responsible for organizing sensations from the body and the environment to produce adaptive and purposeful responses [8]. Sensory integration is thus considered a foundational mechanism for efficient brain function, promoting the connection between the nervous system and behavior [9] and supporting participation in everyday activities [10].

The relationship between sensory stimuli, actions, and behaviors highlights the brain′s ongoing role in interpreting sensory information to generate appropriate responses. This process continues throughout life and becomes increasingly sophisticated as neurological development progresses. Thus, a reciprocal relationship is established, in which the maturation of the nervous system depends on continuous and progressively complex exposure to sensory input [11].

Sensory processing involves the ways in which the central and peripheral nervous systems manage sensory information, including the reception, modulation, integration, and organization of sensory stimuli and the resulting behavioral responses [12]. When sensory processing is inefficient, stimuli may be underestimated, misinterpreted, or elicit responses that are poorly matched to contextual demands, resulting in maladaptive behavioral and functional outcomes [4, 13–15].

This atypical functioning was termed sensory integration dysfunction by Jean Ayres in her early work on sensory integration and has been described as negatively affecting functional performance, including basic activities of daily living (ADLs) [7, 10]. Ayres further proposed that the etiology of sensory integration dysfunction is multifactorial, involving genetic, neurological, and environmental contributors [16].

Building on Ayres′ foundational work, subsequent authors sought to further characterize patterns of atypical sensory functioning. Notably, Miller et al. proposed a nosological classification that organized sensory integration difficulties into three primary categories: sensory modulation disorder, sensory discrimination disorder, and sensory‐based motor disorder; the latter encompassing dyspraxia and postural disorder [17]. Within this model, sensory modulation disorder refers to difficulties regulating responses to sensory input, often manifesting as hyperresponsiveness or hyporesponsiveness; sensory discrimination disorder involves impairments in accurately identifying and interpreting sensory stimuli; and sensory‐based motor disorder reflects challenges in planning and executing purposeful actions [9, 17].

Although this classification has been widely used to describe patterns of atypical sensory functioning, contemporary frameworks in sensory integration, particularly those grounded in the Ayres Sensory Integration (ASI) approach, have progressively shifted away from strict diagnostic categorizations. Current perspectives emphasize a dimensional and functional understanding, in which atypical sensory functioning is described in terms of sensory processing difficulties or differences in modulation, discrimination, and praxis that exist along a continuum of severity and interact dynamically with environmental demands and task characteristics [9]. This approach aligns with occupational therapy′s focus on function, participation, and contextualized performance, as well as with contemporary perspectives on neurodevelopmental conditions, including autism, which recognize variability.

Difficulties in sensory processing can significantly restrict children′s participation in daily activities, negatively affecting overall development and the construction of autonomy [18]. In children with ASD, these challenges are particularly pronounced, as sensory integration difficulties and praxis deficits are well‐documented in this population and have been shown to directly affect performance in ADLs and occupational participation [5, 19, 20].

Sensory processing difficulties in ASD frequently co‐occur with impairments in executive and cognitive functions, including attention regulation, inhibitory control, and memory processes, which compromise learning [21]. These sensory‐related challenges have also been associated with lower levels of functional independence, greater clinical severity, and increased engagement in challenging behaviors, indicating a pervasive influence across developmental domains [5, 22].

Collectively, these difficulties affect not only the child′s functional performance but also the quality of life of the child and their family, as they hinder the ability to carry out routine self‐care tasks such as eating, bathing, dressing, and managing personal and toileting hygiene [23].

According to the World Health Organization [24], participation in daily life activities is a central component of health. For such participation to be effective, individuals must be able to appropriately process and modulate sensory stimuli [7]. This ability is essential not only for engagement in daily routines but also for cognitive development and the learning process [10].

In this regard, studies have shown that ASD severity, along with cognitive impairments, communication difficulties, and sensory processing challenges, is associated with limitations in performance in ADLs [25–27]. Although previous research has explored the development of functional skills in autistic children [28, 29], further investigation is needed to better understand the specific contribution of sensory processing difficulties to occupational performance in ADLs, given their relevance to clinical occupational therapy practice.

Given that independence in daily activities is a top priority for families [19, 30], addressing this knowledge gap requires understanding functional autonomy through the evaluation of sensory processing difficulties from caregivers′ perspectives [6].

In this context, the present study is aimed at contributing to occupational therapy practice by investigating how sensory processing difficulties affect the performance of autistic children in ADLs, providing a foundation for more effective and evidence‐based intervention planning.

## 2. Methods

### 2.1. Study Design and Ethical Approval

This is a cross‐sectional, descriptive study with a qualitative design. This study received ethical approval from the university ethics committee where the research was conducted (Approval No. 6.142.479). All participants signed the informed consent forms prior to data collection.

### 2.2. Participants

Family caregivers were recruited through referrals from occupational therapists at a private clinic in Brazil. Inclusion criteria included being a family caregiver of a child aged 3–8 years with a formal ASD diagnosis confirmed by medical documentation and evidence of sensory processing difficulties in the child, as identified by the Sensory Profile 2 (SP‐2).

The inclusion of caregivers was limited to those responsible for children between 3 and 8 years of age. This age range was selected because children above 3 years typically begin to demonstrate greater independence in daily activities, which enables caregivers to observe and report on their occupational performance more accurately. Children older than 8 years were not included, as they are more likely to have undergone extended therapeutic interventions, which could reduce the observable impact of sensory processing difficulties on daily functioning.

### 2.3. Procedures

Before beginning data collection, the primary researcher completed training on the administration and scoring of the SP‐2 to ensure its accurate application.

Caregivers who met the inclusion criteria were invited to participate in an individual meeting at the clinic where their child received services, between February and July 2024. At first, caregivers responded to the SP‐2 questionnaire, which was administered by the researcher. The SP‐2 is a set of scales that provides information about a child′s responses to different types of sensory stimuli [31], and it is one of the most widely used assessment tools in children with ASD [5]. This standardized instrument, developed by Dunn [32] and adapted for Brazilian Portuguese by Almohalha [33], assesses sensory processing patterns and their effects on children′s participation in everyday activities. The version used in this study included 86 items addressing various domains: auditory, visual, tactile, movement, body position, and oral sensory processing, as well as behavioral, emotional, and attentional responses. Caregivers reported the frequency of behaviors observed in daily life.

A total of 15 children were assessed using the SP‐2, and all were identified as having atypical sensory processing according to the instrument′s scoring system. The sensory profile was administered to ensure that participants met the study′s inclusion criteria, given the absence of a formal medical diagnosis for sensory integration dysfunctions. Consequently, their caregivers met the eligibility requirements and were invited to participate in the interview phase. Of these, 12 caregivers accepted the invitation and completed the interview. Each caregiver received a brief written report summarizing their child′s sensory profile.

A semistructured interview was scheduled and conducted by the first author at a date and time chosen by each participant, with an average duration of approximately 20 min. The interview guide was developed based on theoretical frameworks related to sensory integration and functional performance in ADLs. To ensure consistency and analytical clarity, the questions were grouped into thematic domains, focusing on sensory responsiveness and the child′s autonomy in daily routines. Table 1 summarizes these domains and provides sample questions. The full version of the interview guide is available from the authors upon reasonable request.

**Table 1:** Domains and sample questions from the semistructured interview guide.

Domain	Sample questions
Sensory responsiveness	• Does the child show indifference to heat or cold? • Does the child avoid physical contact? • Does the child get bothered by specific sounds or cover their ears?
Feeding and oral responses	• Does the child refuse food based on texture, color, or smell? • Can the children feed themselves?
Dressing and clothing tolerance	• Does the child accept different types of clothing? • Are there fabrics or tags that cause discomfort? • Can they handle zippers or buttons?
Personal hygiene	• Does the child tolerate nail cutting or hair washing? • Can they brush their teeth independently? • Do they accept specific hygiene products?
Toileting	• Is the child toilet‐trained? • Do they notice when the diaper is dirty? • Are they afraid of sitting on the toilet?
Interaction with textures	• Does the child enjoy walking barefoot? • Are there floor surfaces or materials that they avoid touching?
Vestibular and movement behaviors	• Is the child constantly moving, jumping, or spinning? • Do they show poor awareness of danger?
General autonomy in ADLs	• Can the child perform bathing tasks independently? • Do they need help to dry themselves or use a sponge?

All interviews were audio‐recorded using a mobile device and later transcribed verbatim, ensuring the confidentiality of participants′ identities.

### 2.4. Data Analysis

The interviews were analyzed using content analysis, as proposed by Bardin [34], with the support of MAXQDA software. The process involved three main steps: (1) pre‐analysis, which included an initial reading and organization of the material; (2) exploration of the data, through identification and coding of recording units; and (3) treatment of results, involving the grouping of data into categories based on the principles of mutual exclusivity, homogeneity, relevance, objectivity, and productivity. These steps culminated in the interpretation of the caregivers′ narratives.

## 3. Results

Twelve interviews were conducted with caregivers, including 10 mothers and 2 fathers. The children were predominantly male (*n* = 11), with a mean age of 5.4 ± 1.3 years. Caregivers presented diverse educational backgrounds. All children received an ASD diagnosis at or before 3 years of age and had a consistent history of therapeutic follow‐up. Occupational therapy was reported for all participants, alongside frequent multidisciplinary interventions, including speech‐language therapy, applied behavior analysis‐based interventions, psychology, physiotherapy, and other complementary therapies. Detailed sociodemographic, clinical, and therapeutic characteristics of caregivers and children are presented in Table 2.

**Table 2:** Sociodemographic, clinical, and therapeutic characteristics of caregivers and children.

Participant	Caregiver	Caregiver age (years)	Caregiver educational level	Child sex	Child age at data collection	Age at diagnosis	Therapeutic services
**P1**	Mother	35	High school completed	Male	4 years and 6 months	2 years	Occupational therapy; speech‐language therapy; ABA‐based intervention (multidisciplinary team)
**P2**	Mother	33	Higher education completed	Male	4 years and 7 months	2 years	Occupational therapy; speech‐language therapy; ABA‐based intervention (multidisciplinary team); physiotherapy
**P3**	Mother	43	Elementary school completed	Male	3 years and 8 months	1 year and 6 months	Occupational therapy; speech‐language therapy; ABA‐based intervention (multidisciplinary team)
**P4**	Mother	37	Postgraduate degree	Male	4 years and 2 months	3 years	Occupational therapy; speech‐language therapy; ABA‐based intervention (multidisciplinary team); educational psychology
**P5**	Mother	36	Higher education completed	Male	5 years and 1 month	2 years	Occupational therapy; speech‐language therapy; educational psychology; music therapy
**P6**	Mother	34	Postgraduate degree	Female	5 years and 8 months	2 years	Occupational therapy; educational psychology
**P7**	Mother	36	Higher education completed	Male	4 years and 11 months	2 years	Occupational therapy; speech‐language therapy; ABA‐based intervention (multidisciplinary team)
**P8**	Mother	31	Higher education completed	Male	7 years and 6 months	3 years	Occupational therapy; ABA‐based intervention (multidisciplinary team)
**P9**	Father	29	Higher education completed	Male	6 years and 5 months	2 years	Occupational therapy; speech‐language therapy; ABA‐based intervention (multidisciplinary team)
**P10**	Mother	37	Higher education completed	Male	6 years and 6 months	2 years	Occupational therapy; speech‐language therapy; ABA‐based intervention (multidisciplinary team); educational psychology
**P11**	Mother	45	High school completed	Male	4 years and 7 months	2 years	Occupational therapy; speech‐language therapy; ABA‐based intervention (multidisciplinary team); educational psychology
**P12**	Mother	44	High school completed	Male	7 years and 6 months	3 years	Occupational therapy; psychology; physiotherapy

Abbreviation: ABA, applied behavior analysis.

Sensory processing patterns of the sample were further characterized using the SP‐2. Table 3 presents the distribution of participants across quadrant patterns, sensory sections, and behavioral sections assessed by the instrument.

**Table 3:** Sensory Profile 2 classification of the sample according to quadrant patterns, sensory sections, and behavioral sections (*n* = 12).

Domain	Category	Elevated scores (more or much more than others) *n* (%)
Quadrant patterns	Seeking	10 (83%)
	Avoiding	8 (67%)
	Sensitivity	11 (92%)
	Registration	11 (92%)
Sensory sections	Auditory	5 (42%)
	Visual	5 (42%)
	Touch	11 (92%)
	Movement	9 (75%)
	Body position	6 (50%)
	Oral	9 (75%)
Behavioral sections	Conduct	9 (75%)
	Social‐emotional	9 (75%)
	Attentional	11 (92%)

*Note:* Elevated scores correspond to classifications of “more than others” or “much more than others” according to Sensory Profile 2 normative criteria.

The analysis of interviews allowed the identification of five thematic categories: bathing, feeding, dressing, personal hygiene, and toilet hygiene. This categorization allowed for a structured examination of how sensory integration difficulties impact children′s daily lives, enabling a focused analysis of each domain and its associated consequences.

### 3.1. Bathing

All caregivers reported difficulties their children experienced during bathing. Nine caregivers described the children′s limited coordination and difficulty executing movements required for bathing, such as scrubbing the body or adjusting posture while handling soap or water.

These challenges reflect core characteristics of somatodyspraxia, a type of praxis difficulty characterized by impairments in ideation and motor planning, that is, difficulties in understanding how to organize and execute body movements efficiently, associated with altered processing of tactile, proprioceptive, and vestibular information. These difficulties compromise body awareness and movement sequencing, resulting in poorly coordinated, slow, or inefficient motor actions, with a significant impact on functional performance and participation in ADLs.

“We carefully show and guide him again. Then we try to let him do it, but he still lacks the coordination to do it independently.” (P3)

Two caregivers also described complete dependence on adult assistance during the bathing routine, not only due to motor difficulties but also because the children did not initiate or organize the activity independently. These reports may indicate broader challenges in ideation and task planning, as children struggled to understand the sequence of steps involved or failed to use bathing‐related objects in a functional manner.

Complaints consistent with bilateral integration and sequencing deficit (BISD) were also identified in the reports of three caregivers, describing children′s difficulties in sequencing the movements required to complete tasks, which resulted in increased reliance on assistance.

BISD is a praxis‐related difficulty characterized by impairments in coordinating both sides of the body and sequencing movements efficiently, associated with altered processing of vestibular and proprioceptive sensory information. Functionally, this results in difficulties using the two sides of the body together in an organized and timely manner during daily activities. Such challenges may lead to poorly timed, disorganized, or effortful motor actions, particularly in tasks requiring bilateral coordination and sequential motor planning, with a consequent impact on functional performance and participation in ADLs.

Three children were reported to prefer bathing in a seated position, often using a basin or bucket. Although this behavior alone does not confirm vestibular processing issues, it may suggest postural insecurity, especially in contexts involving wet, slippery surfaces. Seated bathing may serve as a compensatory strategy to minimize environmental demands, possibly reflecting challenges in vestibular modulation or postural control.

“He likes to bathe in a basin. So I fill a basin with water and put some toys for him, and he stays there [sitting], playing.” (P7)

In addition, six caregivers identified behaviors suggestive of tactile hyperresponsiveness, particularly in response to sensations involved in bathing. Children exhibited strong aversive reactions to the texture of sponges or to tactile stimuli during washing and drying, often pulling away or expressing discomfort when touched. These responses are consistent with exaggerated sensitivity to touch, which interferes with engagement in self‐care activities.

“He does not like the sponge; he wants to pull his foot away immediately.” (P1)

### 3.2. Feeding

All 12 caregivers reported that their children experienced difficulties in performing feeding tasks independently and effectively. Two main domains were affected: oral sensory processing and motor planning. Most caregivers (*n* = 9) described behaviors consistent with oral tactile hyperresponsiveness, including strong aversions to specific food textures and refusal to use utensils. These reactions reflect sensory hyperreactivity, leading to restricted food repertoires and disrupted mealtime routines.

“He refuses solid food; he only eats pureed food.” (P2)

Olfactory hypersensitivity was also reported by two caregivers as a factor that interfered with feeding. Caregivers described that food odors triggered marked discomfort, leading to refusal and behavioral outbursts.

“Yes, he smells it and refuses.” (P5)

Two caregivers also reported visual hypersensitivity, noting that the color or presentation of the meal, particularly when involving mixed or contrasting colors, interfered with food acceptance. This restriction in accepted food types impacted the child′s overall dietary variety and participation during meals.

“He refuses food because of the colors.” (P8)

These findings highlight how atypical sensory modulation, specifically hypersensitivity to olfactory and visual input, can negatively impact mealtime engagement.

In addition to sensory issues, motor difficulties also affected feeding performance. Several children (*n* = 6) required constant supervision or physical assistance for basic tasks such as bringing food to the mouth or using cutlery. These observations are compatible with somatodyspraxia, marked by poor body awareness, initiation deficits, and impaired motor coordination.

“We have to feed him. We need to put the food on the spoon. Once the food is on the spoon, he can bring it to his mouth. But making the movement to scoop food from the plate, he doesn′t do that.” (P1)

Signs of ideational dysfunction were also observed in three children, including difficulties understanding what to do with the food (e.g., placing it in the mouth without chewing). BISD further contributed to feeding dependence on one child, who was unable to coordinate bilateral motor actions required during feeding.

Moreover, one caregiver described episodes of motor agitation during meals, in which the child frequently moved away from the table or failed to remain seated. Such behaviors may suggest vestibular hyporesponsiveness, characterized by a high threshold for movement input and reduced postural control, potentially interfering with the continuity and effectiveness of feeding.

### 3.3. Dressing

All children in the study exhibited difficulties in performing dressing tasks. A variety of sensory and motor factors appeared to contribute to these challenges, with varying degrees of impact on autonomy and functional performance.

The most frequently reported sensory issue was tactile hyperresponsiveness, described by nine caregivers as discomfort related to specific textures and clothing tags.

“Clothing tags bother him. Sometimes, if there′s a tag, he keeps holding it for a while.” (P2)

In addition, nine caregivers reported that their children resisted wearing winter clothing, especially items that covered the body more extensively. Although the underlying mechanisms were not fully clear, this behavior may reflect sensory modulation difficulties, possibly linked to tactile hyperresponsiveness (discomfort with the feel of heavy or layered fabrics) or to a preference for increased environmental sensory input, which becomes restricted when the body is more fully covered.

“She does feel it, but her need to be without clothes is stronger than the cold.” (P11)

Motor planning difficulties were also prominent, with several caregivers (*n* = 9) reporting that their children required full assistance to complete the dressing routine. These observations align with features of somatodyspraxia, particularly poor motor coordination, limited postural control, and difficulty initiating and sequencing body movements. Such impairments directly compromise the child′s independence in dressing and increase the caregiver′s involvement in daily routines.

“We dress her. Because she stiffens up a lot, and movements like raising her arms bother her a lot.” (P11)

Some caregivers (*n* = 3) also described challenges that may be related to bilateral integration difficulties, such as difficulty coordinating both sides of the body or maintaining postural stability during dressing tasks. Although these signs were not always described in detail, they suggest the need for further evaluation of potential BISD.

Finally, difficulties in organizing the dressing sequence were noted by three caregivers. These behaviors may reflect challenges in motor planning and, in some cases, could suggest emerging ideational aspects, such as difficulty understanding the sequence of dressing steps or selecting effective strategies to complete the task. However, such interpretations should be made cautiously, as the behaviors described may also relate to bilateral integration and sequencing challenges. In some instances, they may simply represent atypical but functional dressing routines.

“Always the hardest way. He puts one arm in first, then the head, then the other arm.” (P9)

### 3.4. Personal Hygiene

Personal hygiene was reported as a particularly challenging activity by 11 caregivers. This category was subdivided into two key areas: toothbrushing and hair and nail cutting.

Regarding toothbrushing, most caregivers (*n* = 10) described strong behavioral and emotional reactions during the activity. The most frequently reported difficulty was oral tactile hyperresponsiveness, manifested as aversion to the presence of the toothbrush in the mouth or to the taste and texture of toothpaste. Caregivers indicated that these reactions were associated with marked difficulty engaging in the task.

“Brushing his teeth every day is chaos, like we′re hurting him.” (P3)

Sensory processing difficulties were frequently accompanied by motor planning challenges, resulting in the need for full assistance in five children. These cases were described in terms of caregivers having to perform the entire brushing sequence for the child, which may reflect motor planning impairments consistent with somatodyspraxia. In these instances, children appeared unable to plan or execute the complex movements needed to brush and rinse effectively.

“I have to brush them. I rinse too.” (P5)

Some caregivers (*n* = 3) reported that children were encouraged to attempt toothbrushing independently, but their performance was ineffective and required adult correction. Although these behaviors could stem from developmental immaturity or general motor delays, they may also indicate early signs of bilateral coordination challenges, particularly in tasks that demand the coordinated use of both hands and symmetrical movement patterns. However, given the limited detail in these reports, such interpretations should remain hypothetical

“We let him try on his own, but then we have to brush again because he doesn′t do it properly yet.” (P8)

Two caregivers described behaviors that could be interpreted as possible signs of ideational difficulty, such as not understanding the purpose or function of the toothbrush. For example, biting the brush instead of using it appropriately may suggest difficulty conceptualizing the task. However, such behaviors could also be influenced by oral sensory seeking tendencies or general unfamiliarity with the routine and should not be interpreted as definitive evidence of ideational difficulty.

“He bites the toothbrush, he can′t do it. I have to hold his little hand to guide him.” (P6)

With regard to haircuts and nail trimming, nine caregivers reported difficulties strongly associated with tactile hyperresponsiveness. Children exhibited marked aversive reactions to these activities, including resistance, agitation, and occasionally, disruptive behaviors. Some caregivers reported that the procedures could only be performed while the child was asleep, underscoring the severity of sensory discomfort.

“Only when he′s asleep, he won′t let us cut his nails otherwise.” (P6)

### 3.5. Toilet Hygiene

Toilet hygiene was reported as a challenge by 11 caregivers. The difficulties observed were associated with a range of sensory modulation issues, particularly related to tactile and vestibular processing, followed by challenges in motor planning and execution.

Two caregivers described tactile hyperresponsiveness in children, characterized by strong discomfort associated with being soiled. In these cases, children reacted immediately to sensations of dirtiness by requesting a diaper change, a bath, or other forms of cleaning.

“When he poops, he immediately calls to go to the bathroom because he wants a bath.” (P1)

In contrast, five caregivers described behaviors suggestive of reduced tactile awareness, such as not perceiving the need to use the toilet or not noticing when they were already soiled. These behaviors may be related to tactile discrimination difficulties, although cognitive and behavioral factors could also contribute.

“He stays in a dirty diaper… he doesn′t notice.” (P10)

Additionally, two caregivers mentioned that their children had difficulty remaining seated on the toilet, which interfered with the continuity of the activity. This behavior may reflect vestibular hyporesponsiveness, potentially linked to poor postural control or a constant need for movement input.

Turning to motor aspects, four caregivers also reported difficulties consistent with somatodyspraxia, characterized by challenges in body awareness, motor planning, and the execution of purposeful movements required during hygiene routines. In these cases, children required full assistance to perform tasks such as wiping after elimination.

Additionally, signs of BISD were observed in three children who struggled to coordinate both sides of the body effectively. These difficulties were particularly evident in tasks requiring alternating bilateral movements, such as managing toilet paper, which further compromised their independence in toileting.

“When he′s done, he calls me to wipe him.” (P8)

Together, these sensory and motor challenges significantly interfered with the development of autonomy in toilet hygiene. The findings highlight the importance of individualized support strategies that address both sensory processing and motor planning needs in this daily activity.

To provide a structured summary of the sensory and motor patterns identified across the five thematic categories, Table 4 presents the characteristics derived from the analysis of caregiver narratives and organized within sensory processing domains based on ASI theory.

**Table 4:** Sensory and motor difficulties identified through qualitative analysis of caregiver narratives across ADLs.

Sensory processing domain	Sensory and motor difficulties	Bathing	Feeding	Dressing	Personal hygiene	Toilet hygiene
Praxis	Somatodyspraxia	•	•	•	•	•
	Ideational difficulties	•	•	•	•	
	Bilateral integration and sequencing deficit	•	•	•	•	•

Sensory modulation	Tactile hyperresponsiveness	•		•	•	•
	Oral tactile hyperresponsiveness		•		•	
	Olfactory hypersensitivity		•			
	Visual hypersensitivity		•			
	Postural insecurity	•				
	Vestibular hyporesponsiveness		•			•

Sensory discrimination	Reduced tactile awareness					•

Overall, the analysis revealed that sensory and motor difficulties were identified across all five ADLs, with overlapping patterns spanning praxis, sensory modulation, and sensory discrimination domains.

## 4. Discussion

The world is fundamentally sensory in nature [11]. Individuals are continuously exposed to stimuli from multiple sensory modalities, requiring an efficient central nervous system to interpret, discriminate, integrate, and organize this information [11, 35]. This process, termed sensory integration [7], enables appropriate motor and behavioral responses critical for occupational performance. Consequently, impairments in sensory integration may lead to dysfunctions [18] that can directly affect ADLs.

Motor function emerges early in childhood, with children progressively internalizing praxis, the interface between cognitive and motor processes that enables effective interaction with the environment [36]. Praxis encompasses ideation, motor planning, and motor execution. Dyspraxia is a praxis difficulty impacting one or more of these components, manifesting as difficulty in spatial organization and motor sequencing [36]. Jean Ayres (1970) notably linked praxis to sensory integration, emphasizing that sensory dysfunctions hinder the development of body‐related skills crucial for children′s participation and functional independence in daily activities [37].

In this study, praxis difficulties were prevalent, with somatodyspraxia emerging as the dominant pattern. This condition is characterized by impaired motor coordination alongside tactile processing difficulties, resulting in broader functional limitations [10]. Observed limitations in body awareness, motor planning, and postural adaptation may compromise performance across all investigated ADLs, including bathing, dressing, feeding, personal hygiene, and toileting. These findings align with prior research highlighting the frequent occurrence of praxis difficulties in children with autism and their impact on daily life experiences [38–40].

Additionally, some caregivers reported behaviors suggestive of BISD, a subtype of praxis difficulty involving impaired coordination of both sides of the body, postural instability, and disrupted motor sequencing. Tasks requiring alternating and symmetrical movements, such as manipulating clothing fasteners or using toilet paper, are described as particularly challenging for autistic children with praxis difficulties [41]. These observed challenges may be associated with vestibular and proprioceptive processing inefficiencies characteristic of BISD [40]. However, these interpretations should be considered initial clinical hypotheses, derived from caregiver reports, and require confirmation through specialized occupational therapy assessments.

Ideation, the ability to conceptualize objects and their functions, is the initial stage of motor planning preceding action execution [42]. Children with ideational deficits may struggle to understand what to do, how to use objects, and how to organize task steps mentally [36, 42]. Given that ADLs require sequential actions to be successfully completed [43], impaired ideation may compromise performance. For instance, children may demonstrate difficulty identifying bathing steps, holding food without chewing, employing disorganized dressing strategies, or misunderstanding toothbrushing purposes. These functional repercussions are consistent with literature exploring the impact of praxis difficulties on daily activities [44, 45].

Sensory modulation difficulties were widely reported by caregivers, affecting all sensory modalities except proprioception and may hinder participation in daily tasks [27]. Such difficulties may provoke disproportionate behavioral responses, resulting in avoidance and increased stress, which negatively influence developmental skill maintenance and overall functionality [46, 47].

Tactile hyperresponsiveness was the most frequent sensory modulation issue, identified in caregivers′ reports, interfering with self‐care tasks such as bathing (rejection of sponges, water, and hair washing), feeding (texture refusals), dressing (discomfort with clothing tags and fabrics), personal hygiene (aversion to nail/hair cutting and toothbrushing), and toileting (refusal to remain soiled). Conversely, signs of tactile hyporesponsiveness, such as unawareness of wet diapers or dirtiness, were also described, which may complicate autonomy acquisition, especially in toilet training. Prior research corroborates the high prevalence and functional impact of tactile modulation difficulties in this population [48].

Vestibular hyporesponsiveness was inferred from caregiver descriptions of behaviors like difficulty remaining seated and constant movement seeking during posturally demanding tasks (toileting, bathing, and eating). In the literature, these behaviors are interpreted as reflecting impaired vestibular regulation, with children requiring continuous movement input to maintain sensory balance, thereby compromising engagement and independence in these activities [49, 50].

Oral hyperresponsiveness was commonly reported during feeding and toothbrushing, with children exhibiting aversions to specific textures, flavors, or oral sensations. Food refusal is notably prevalent among autistic children and is consistently linked to atypical oral sensory processing [51, 52]. These sensory challenges contribute to limited dietary variety, reluctance to try new foods, and reduced overall intake [51, 53]. Caregivers also noted olfactory and visual hypersensitivities affecting food acceptance, suggesting a relationship between sensory processing difficulties and restrictive eating patterns [54]. Children often prefer predictable sensory food experiences (uniform texture, mild taste, and familiar appearance) as a regulatory strategy, but these preferences can lead to rigid diets and nutritional concerns [55, 56].

Sensory discrimination, the capacity to accurately identify and differentiate sensory stimuli, is essential for effective sensory processing and functional performance [10]. Impairments in this area compromise occupational performance as the brain struggles to interpret sensory input accurately [11, 57]. A recent study assessing somatosensory detection and temporal discrimination thresholds in children with ASD demonstrated associations between these deficits, clinical sensory symptoms, and daily functional difficulties, highlighting their relevance to emotional and behavioral responses [58].

Tactile discrimination difficulties can impact activities such as dressing and toileting. Some children reportedly preferred clothing exposing more skin, which may reflect a compensatory behavior to increase sensory input and improve discrimination [59]. Conversely, others with heightened tactile discrimination rapidly perceived dirtiness and demanded immediate cleaning or diaper changes [40].

It is important to emphasize that sensory processing domains (modulation, discrimination, and praxis) rarely occur in isolation. Rather, they often overlap, interact, and amplify one another, intensifying the barriers to functional engagement and meaningful participation in daily activities [10, 60].

Collectively, caregiver observations indicate that sensory and motor processing difficulties substantially interfere with children′s performance in ADLs, often resulting in increased dependence on caregivers. In families of children with ASD, the need for constant supervision, heightened support, and reduced flexibility in daily routines may negatively influence family life. Evidence indicates that children′s sensory experiences affect not only their functional performance and participation but also the functioning of their families, contributing to increased caregiving demands and emotional burden [6].

Difficulties in the performance of ADLs are consistently associated, in the literature, with intensified daily care routines and higher levels of caregiver stress, which increase vulnerability to mental health conditions such as depression and anxiety [61]. Furthermore, the quality of life of families caring for children with neurodevelopmental disorders is shaped by multiple factors, including the severity of the condition, the availability of family and social support, and access to specialized services. Parental stress, in particular, has been shown to affect family dynamics and overall quality of life due to the additional demands of caregiving, especially those related to supporting children′s participation and independence in daily activities [62].

Taken together, the findings of the present study, interpreted in light of existing evidence, suggest that the impact of sensory processing difficulties on children′s performance in daily activities and the resulting increase in family stress associated with intensified caregiving demands highlight the need for evidence‐based occupational therapy interventions aimed at reducing sensory‐related challenges in children with ASD.

Occupational therapy interventions for autistic children commonly incorporate sensory‐rich activities tailored to each child′s individual needs, with the aim of promoting adaptive responses and supporting participation in meaningful daily activities [63]. Evidence indicates that occupational therapy interventions can provide substantial benefits for autistic children, facilitating engagement in essential activities that were previously limited by sensory processing difficulties [64, 65].

Among sensory‐based approaches, interventions grounded in the ASI framework have received the strongest empirical support. A recent systematic review [12] identified multiple therapeutic approaches applying sensory integration principles in the treatment of children with ASD and demonstrated significant improvements in behavior and daily performance. Additional benefits were observed in communication and socialization. These findings are consistent with previous systematic reviews concluding that ASI constitutes an evidence‐based practice for children with ASD [66, 67]. Notably, some studies have also reported meaningful changes in children′s play patterns following sensory integration interventions, highlighting the broader impact of these approaches on occupational engagement.

Despite these positive outcomes, the literature also highlights important limitations. Considerable heterogeneity in intervention protocols, outcome measures, and study designs restricts the generalizability of findings and underscores the need for greater standardization of intervention methods. Furthermore, variations in methodological rigor across studies indicate that additional high‐quality research is required to clarify optimal dosage, implementation parameters, and the applicability of sensory integration interventions across diverse clinical and nonclinical settings [12].

Taken together, current evidence supports the use of occupational therapy interventions grounded in sensory integration principles as an effective strategy for improving functional performance and participation in daily activities among autistic children. These findings reinforce the importance of evidence‐based sensory interventions within occupational therapy practice while also highlighting the need for continued research to refine intervention protocols and strengthen clinical recommendations.

As a limitation of the present study, the findings are based exclusively on caregiver reports, which, although valuable for capturing real‐world experiences, may introduce subjective bias and limit generalizability. Additionally, caregivers′ reports may have been influenced by parental stress levels and by variations in understanding technical concepts related to functional performance, such as distinctions between independent and atypical task execution. In addition, participants were recruited from a single private rehabilitation clinic in Brazil, which may reflect a specific socioeconomic context characterized by greater access to specialized services and therapeutic resources. This context may have influenced caregivers′ perceptions, expectations, and interpretations of their children′s sensory and functional challenges. Therefore, the findings should be interpreted with caution and may not be fully generalizable to families receiving care in public health services or to different cultural and healthcare settings. Future research would benefit from mixed‐methods approaches, broader and more diverse samples, and the incorporation of standardized assessments and direct observations to enhance clinical rigor and strengthen the applicability of findings.

## 5. Conclusions

This study highlights the significant impact of sensory processing difficulties on the daily functioning of autistic children, demonstrating how these challenges alter the perception, interpretation, and organization of sensory input and interfere with independent participation in ADLs. The findings indicate that sensory‐related challenges shape everyday experiences in ways that directly compromise functional autonomy and occupational engagement.

Across all cases examined, essential elements required for task execution were affected, with sensory modulation difficulties, particularly patterns of hyperresponsiveness and hyporesponsiveness, pervasively influencing performance in basic ADLs and frequently co‐occurring with praxis‐related difficulties. These results underscore the complex and multifaceted nature of sensory processing challenges and their cumulative impact on children′s functional independence.

Taken together, the findings reinforce the importance of individualized occupational therapy interventions that address specific sensory and motor planning needs to support participation, autonomy, and engagement in daily routines. From a clinical perspective, understanding how sensory processing difficulties manifest in everyday activities may guide more targeted and meaningful intervention planning.

As a limitation, data collection relied on caregiver reports from families recruited in a single private clinical setting, which, although valuable for capturing real‐life experiences, may introduce subjective bias and limit generalizability of the findings to public health services or other cultural and socioeconomic contexts. Future research would benefit from broader methodological approaches, including standardized assessments and direct observation. Nevertheless, by amplifying family perspectives, this study provides clinically relevant insights into the everyday impact of sensory processing difficulties and reinforces the role of occupational therapy in addressing both sensory and functional demands in the lives of autistic children.

## Author Contributions

L.S.T. contributed to the study design, data collection and analysis, manuscript writing, and final revision. B.B.W. contributed to data collection and analysis and the final revision of the manuscript. A.M.P.S. contributed to the study design, data analysis, manuscript writing, and final revision.

## Funding

This study was supported by São Paulo Research Foundation (FAPESP) (Grant Number: 2024/07114‐6).

## Conflicts of Interest

The authors declare no conflicts of interest.

## Data Availability

This is a qualitative study; the transcripts are not publicly available.
